# Hemorrhagic Transformation in Patients With Ischemic Stroke and Atrial Fibrillation: To Anticoagulate or Not, That Is the Question

**DOI:** 10.7759/cureus.53548

**Published:** 2024-02-04

**Authors:** Tiago Vasconcelos, Fábio Caleça Emidio, Frederico Silva, Joana Nascimento, Marta Duarte

**Affiliations:** 1 Internal Medicine, Centro Hospitalar Universitário do Algarve - Unidade de Portimão, Portimão, PRT; 2 Internal Medicine, Centro Hospitalar Universitário do Algarve - Hospital de Faro, Faro, PRT

**Keywords:** hemorrhagic risk, anticoagulant therapy, hemorrhagic transformation, atrial fibrillation, stroke

## Abstract

The management of anticoagulation in patients with ischemic stroke and atrial fibrillation (AF) poses a critical dilemma due to the inherent risk of hemorrhagic transformation.

This article presents the case of an 89-year-old male with AF and recurrent ischemic strokes, highlighting the complex challenge of deciding whether to initiate or withhold anticoagulation. After the initial ischemic stroke event, the patient started a direct oral anticoagulant. Subsequent imaging revealed hemorrhagic transformation, leading to the cessation of anticoagulation. Despite multiple hemorrhagic recurrences, balancing thrombotic and bleeding risks remained challenging. Mechanical thrombectomy was performed for a subsequent ischemic stroke due to an absolute contraindication for thrombolysis.

The patient's intricate clinical course involved a multidisciplinary approach, resulting in a decision to cautiously resume low-dose anticoagulation combined with left atrial appendage closure. This decision was made after careful consideration of persistent thrombotic risk despite recurrent hemorrhages.

The case underscores the complex management dilemma of anticoagulation in elderly patients with AF and recurrent strokes, emphasizing the need for a multidisciplinary approach and individualized decision-making in such challenging scenarios. Further research and guidelines are warranted to establish optimal strategies for (re)initiating anticoagulation in patients with recurrent hemorrhagic transformation.

## Introduction

Atrial fibrillation (AF) is a common condition with a high prevalence in the elderly population, which is directly proportional to the patient's age. Considering this proportion, in Europe, the incidence of ischemic stroke increases from 1.5%, in patients aged 50 to 59, to 23.5%, in those aged 80 to 89. The cardioembolic stroke, secondary to AF, accounts for approximately 30% of all ischemic strokes [1].

Secondary prevention for ischemic stroke in patients with AF is anticoagulant therapy. However, the main side effect of this treatment is an increased risk of bleeding. Before initiating anticoagulant therapy, it is essential to assess both the hemorrhagic and the embolic risk so that the complications associated with this therapy do not outweigh its potential benefits, with the primary goal of improving the patient's quality of life.

## Case presentation

An 89-year-old male patient, independent in activities of daily life, with a previous medical history of transient ischemic attack in 2016 and 2018 of undetermined etiology and dyslipidemia, is currently medicated with acetylsalicylic acid (aspirin) and a statin.

**Figure 1:**
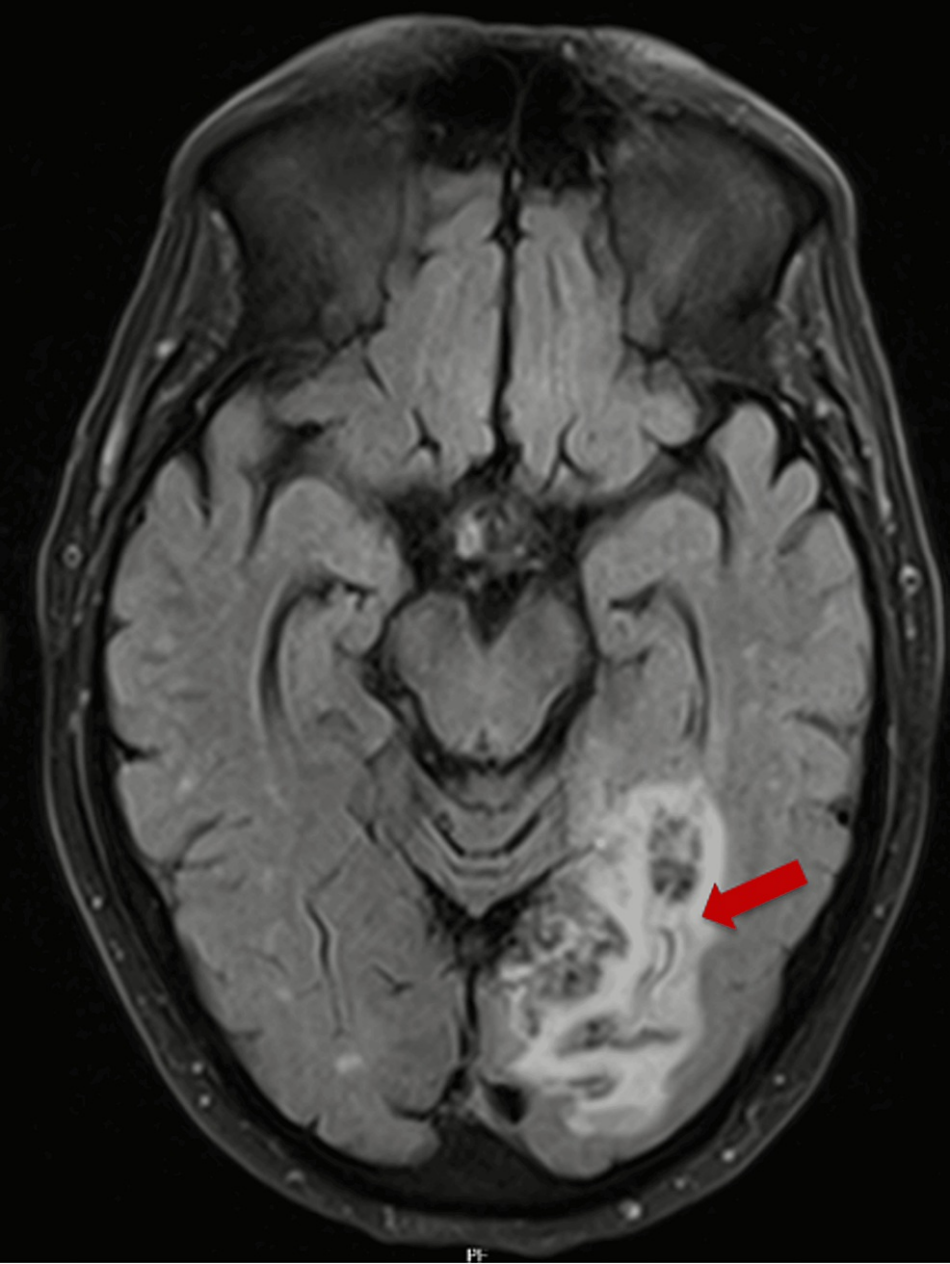
MRI presenting an ischemic lesion with hemorrhagic transformation (indicated by a red arrow)

In June 2021, the patient sought the emergency department (ED) because he was experiencing difficulty in his right-sided vision. He was assessed by the physician who diagnosed the right homonymous hemianopsia and performed the National Institutes of Health Stroke Scale (NIHSS), scoring 1 point. Among the complementary exams performed in the ED, the electrocardiogram highlighted AF with rapid ventricular response. Imaging-wise, on computed tomography (CT) and CT angiography of the head and neck, there was noted “hypodensity of the white matter, in the occipital and medial posterior temporal regions on the left, with a mass effect,” which could indicate a primary neoplastic lesion or acute/subacute ischemic vascular lesion. During hospitalization, further imaging with MRI of the brain confirmed the presence of an ischemic region with hemorrhagic transformation in the same region as noted on the initial CT scan, without signs of amyloid angiopathy (Figure 1).

With no worsening of neurological deficits observed, a contrast-enhanced CT scan was performed on the sixth day to establish a plan regarding the antithrombotic strategy to be instituted. The CT scan showed a hematoma in absorption, with a simultaneous reduction in its size. A Holter study and electroencephalogram were also performed, confirming the presence of paroxysmal AF and a normal alpha base rhythm, respectively. On the day the CT scan was performed, the patient started taking edoxaban (due to its once-daily dosage) and was discharged the day after the initiation of the treatment.

Three days after discharge, the patient was readmitted to the ED due to a mechanical ground-level fall with traumatic brain injury, without any new focal neurologic defects other than the right visual field defect from the previous stroke. On objective examination, the patient was normotensive, with a small wound on the occipital scalp. The blood work was normal. The contrast-enhanced CT showed worsening hypodensities suggestive of hemorrhagic transformation in the region of the previously established ischemic lesion, with no evidence of mass effect. Considering the asymptomatic hemorrhagic transformation, the decision was made to suspend anticoagulation and reassess via CT, six days later. The patient showed favorable and expected clinical and imaging evolution, with a reduction in hematoma content. Nevertheless, the choice was made to continue withholding antithrombotic therapy, and a repeat CT was planned after two weeks to evaluate the stability of the hemorrhage, subsequently considering the reintroduction of anticoagulation.

**Figure 2:**
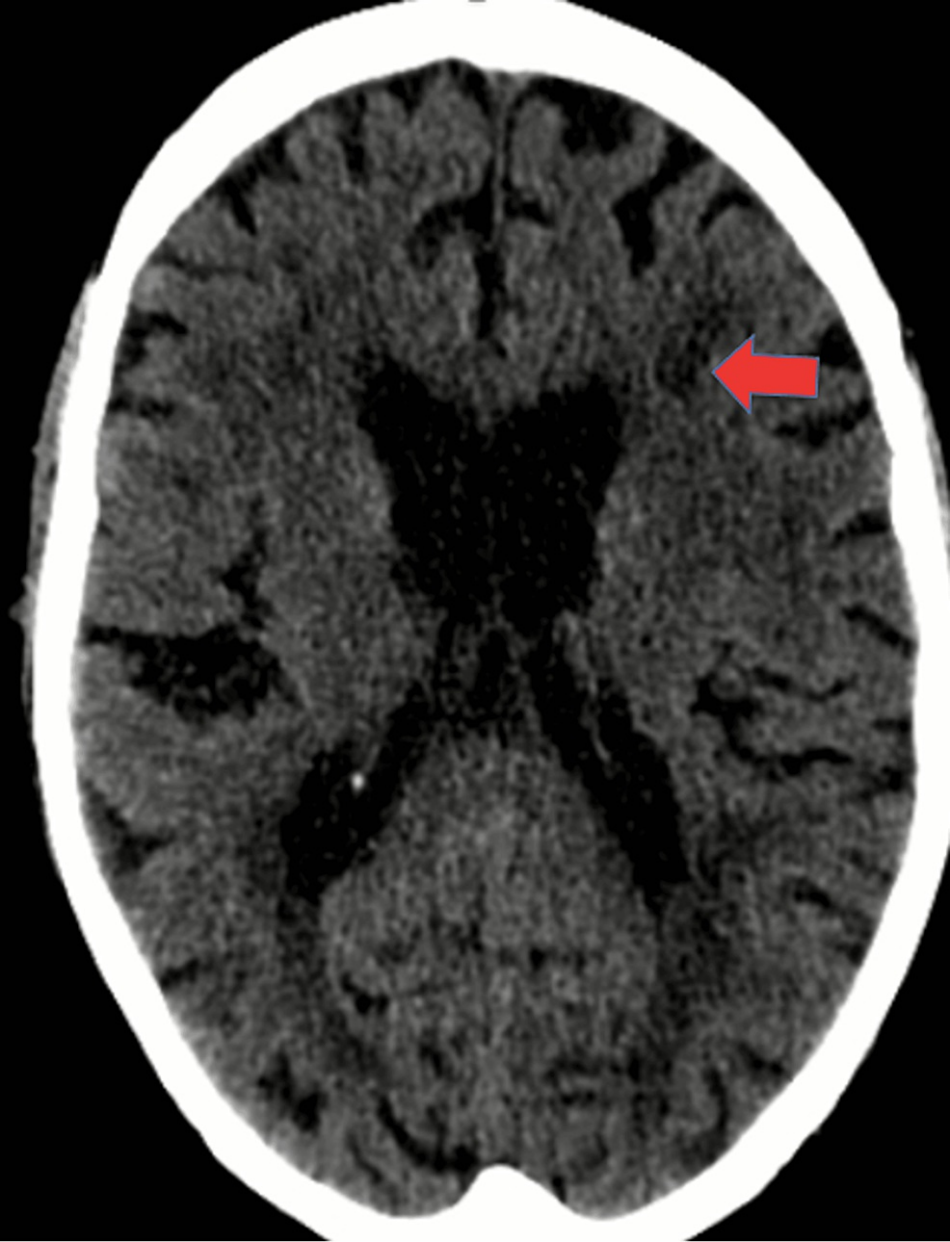
CT revealing the presence of cortico-subcortical insular and frontal hypodensity, indicating recent ischemic injury of the left MCA (indicated by a red arrow)

On the day preceding the appointment, the patient was taken to ED by his daughter after he had a sudden-onset of global aphasia, right homonymous hemianopsia, and right central facial paralysis, with an NIHSS score of 12. Upon admission, he was hemodynamic stable, normoglycemic, and afebrile. A non-contrast CT scan revealed the presence of a hyperdensity in the left middle cerebral artery territory involving the portico-subcortical insular and frontal cortex, indicating ischemic injury (Figure 2).

Cerebral angiography also demonstrated interruption of contrast filling in the proximal upper M2 branch of the left MCA. Considering the imaging findings and the patient's absolute contraindication for thrombolysis (recent intracranial hemorrhage), mechanical thrombectomy was performed. Partial vessel recanalization was achieved (Thrombolysis in Cerebral Infarction (TICI) scale, TICI 2b). He was admitted to the stroke unit and underwent a follow-up CT 24 hours after admission, revealing a left posterior parietal cortico-subcortical infarct with reabsorption of posterior occipital hematoma (region related to the hemorrhagic transformation present in the previous hospitalization).

After three days, a repeated CT scan showed the presence of subcentimeter hemorrhagic foci in the periventricular temporal and high frontal regions, indicating an improvement in imaging. Following this, and after discussing the clinical case with the neurology team and weighing the hemorrhagic and thrombotic risks, the decision was made to resume anticoagulation. After initiating antithrombotic therapy, a repeat CT, on the third day, demonstrated new left frontoparietal and interhemispheric frontal hemorrhagic foci without neurological worsening, resulting in the suspension of this therapy. A new CT scan three days after suspension showed stability of the hemorrhagic component. During the hospitalization in the stroke unit, there was a favorable clinical evolution, with a score of 5 points (right homonymous hemianopsia, right central facial paralysis, and mild dysarthria) on the NIHSS at the time of discharge.

After a multidisciplinary discussion of this clinical case involving internal medicine, neurology, and cardiology, it was decided to initiate anticoagulant therapy (60mg of edoxaban), maintaining neurological stability up to the present date. Closure of the left atrial appendage was also proposed, after a cardiology assessment, complemented with contrast-enhanced echocardiography. A transesophageal echocardiogram was conducted to assess the presence of intracardiac thrombi. The results confirmed the absence of thrombi in the left atrial appendage and revealed a favorable anatomy for occlusion procedures. Following the completion of this study, closure of the atrial appendage was performed and anticoagulant therapy was maintained for six weeks. After the closure of the atrial appendage, the patient remained clinically stable from a neurological standpoint, with no new thrombotic or hemorrhagic complications, up to the present date.

## Discussion

Antithrombotic therapy plays a crucial role in the secondary prevention of cardioembolic etiology strokes, particularly in patients with atrial AF, one of the leading causes of such events [1]. Early diagnosis of AF is essential, given its prevalence and association with strokes [1].

Current recommendations emphasize the need for prolonged cardiac monitoring to exclude cardioembolic causes in cases of transient ischemic attacks or strokes with undetermined causes. Additionally, implantable devices are suggested for detecting subclinical AF [2]. This patient could have benefited from linq monitoring (implantable cardiac event monitor) as his previous transient ischemic attacks did not reveal a clear etiology.

While antithrombotic therapy is associated with an increased risk of bleeding, the presented clinical case underscores that, despite multiple hemorrhagic recurrences, thrombotic risk consistently outweighed bleeding risk. This clinical challenge raises questions regarding the decision to initiate or reintroduce anticoagulation in elderly patients with intracranial hemorrhage, an area lacking comprehensive studies [3].

In this complex clinical scenario, the multidisciplinary decision leaned towards the closure of the left atrial appendage [4]. This approach aims to reduce thrombotic risk without increasing hemorrhagic risk.

These considerations underscore the urgent need for additional research and specific guidelines to establish optimal strategies, including the timing of (re)initiating anticoagulation in patients with recurrent hemorrhagic transformation. In this context, it is noteworthy that recent studies, as published in the American Heart Association journal and others, have explored crucial rules such as the "1-3-6-12" guideline for commencing anticoagulation after ischemic stroke [5]. These studies provide valuable insights into best practices and timelines for anticoagulation initiation, potentially influencing decision-making in cases like the present one. Developing clearer, evidence-based approaches, informed by these studies, is crucial to enhance clinical practice and optimize outcomes in patients with AF and a significant risk of stroke.

## Conclusions

Stroke is the leading cause of death and disability in Portugal. Cardioembolic etiology accounts for a significant portion of these ischemic events, and antithrombotic therapy is considered the cornerstone of its treatment, albeit with associated side effects.

The closure of the left atrial appendage is a therapeutic option to be considered in selected cases, as presented. It is crucial to include patient follow-up data to validate the long-term effectiveness of the treatment approach.
